# Ketamine addiction following a single sub-anaesthetic ketamine treatment for acute suicidality in a psychiatrically multimorbid patient: case report

**DOI:** 10.1192/bjo.2026.11012

**Published:** 2026-04-21

**Authors:** Gijsbrecht H. J. Roelandt, Jurriaan F. M. Strous, Jeanine Kamphuis, Robert A. Schoevers, Radboud M. Marijnissen

**Affiliations:** University Medical Centre Groningen (UMCG), Groningen, The Netherlands; Lentis Mental Health Care, Groningen, The Netherlands

**Keywords:** Addiction, suicide, depressive disorders, acute psychiatry, clinical trials

## Abstract

This case report presents the case of a 25-year-old woman who developed ketamine addiction following a single sub-anaesthetic dose of intranasal ketamine in a pilot study investigating intranasal racemic ketamine for acute suicidality. She had a history of depression, obsessive–compulsive disorder, autism spectrum disorder and anorexia nervosa, and she had sporadically used alcohol and cannabis. Following the intervention, she reported a transient reduction in suicidal ideation but later sought illicit ketamine to recreate its calming effects on intrusive thoughts. Subsequently she also started abusing cocaine and 3-methylmethcathinone (3-MMC). Within weeks she had escalated to daily use, which led to financial distress, housing instability and a suicide attempt when access was cut off. Although she initially ceased use, she later relapsed into ketamine and cocaine addiction. This case highlights the addictive risk of ketamine, even in controlled settings. Given ketamine’s rising use in psychiatric treatment, careful screening, monitoring and awareness of addiction potential are essential. Future research should evaluate patient-specific risk factors and dosing strategies to minimise abuse liability.

In this case report, we present a patient who developed ketamine addiction following a single dose of intranasal ketamine that was prescribed in the context of the ketamine trial for acute suicidality (KETA) pilot study.^1^ This pilot study was conducted before the larger, double-blind, randomised controlled KETA trial, which had the objective of evaluating whether 75 mg of intranasal racemic ketamine would diminish suicidality more than 4 mg of intranasal midazolam, as an active placebo. The pilot study was conducted to assess the feasibility and acceptability of the double-blind, randomised controlled trial (RCT). In the pilot study, a heterogenous group of patients experiencing an acute increase in suicidality received a single, open-label dose of 75 mg intranasal racemic ketamine. Racemic ketamine is a mixture of two enantiomers, R-ketamine and S-ketamine, which are mirror images of each other. Vital functions, adverse effects, mood and suicidality were monitored. Depression scores were measured using the Montgomery–Åsberg Depression Rating Scale (MADRS), and suicidality severity was measured using the Beck Scale for Suicidal Ideation (BSSI). These questionnaires were administered at baseline and at 60 min, 180 min and 1, 3 and 7 days following ketamine administration. Subjects for this study were enrolled from various divisions of the Psychiatry Department of the University Medical Centre Groningen (UMCG) and from Lentis Mental Health Care, Groningen. Written informed consent was obtained from all subjects. The authors assert that all procedures contributing to this work comply with the ethical standards of the relevant national and institutional committees on human experimentation, and with the Helsinki Declaration of 1975 as revised in 2013, which was the most current revision at the time.^2^ All procedures involving human subjects/patients were approved by the Institutional Review Board (IRB) Groningen under approval no. 2020/378. Subjects who expressed interest in participating in the study received written and oral information on the study procedures. Given the acute nature of acute suicidality, and the objective of the KETA study to find a treatment for acute suicidality, subjects had a minimum of 1 h to decide whether they wished to participate in the study. Study procedures would start either the same day or the day after written informed consent was obtained.

Based on results of the pilot study, we designed a double-blind RCT. This case report addresses a serious risk of clinical ketamine use that we believe is important to share with both clinicians and researchers.

## Case presentation

Mrs. S. was, at time of treatment, a 25-year-old woman with a history of recurrent depressive episodes, obsessive–compulsive disorder, autism spectrum disorder and anorexia nervosa, with no history of addiction disorders. The substance abuse anamnesis revealed that her past substance use was limited to sporadic use of alcohol and cannabis, the adult use of these being legal and tolerated in law, respectively, in the Netherlands. This use pattern did not qualify as a substance use disorder. One day before enrolment in the KETA pilot, by means of a crisis intervention for her acute suicidality, she was admitted to the acute in-patient psychiatry ward of the UMCG.

In the past the patient had exhibited recurrent suicidality, reflected by more than ten previous suicide attempts. Various therapeutic interventions were tried to attenuate her depressive symptoms and manage suicidality, namely pharmacotherapeutic interventions: venlafaxine, clomipramine, fluvoxamine, benzodiazepines and individual cognitive–behavioural therapy. Because therapeutic interventions had no lasting effect on her psychiatric multimorbidity and suicidality, she was living in a sheltered psychiatric housing facility. Besides this, acute exacerbations in suicidality or suicide attempts, often auto-intoxications, frequently necessitated admission to a secured psychiatric ward.

Before her admission to the acute in-patient ward in November 2021, due to an acute increase in suicidal ideation. she had been treated at the acute out-patient clinic of the UMCG since March 2021. The exacerbation that led to her admission was triggered by the announcement from her psychiatrist at the UMCG that further care was going to be provided by a different mental healthcare institution, which caused significant emotional distress. Later that day the police found her wandering near railway tracks, and brought her to the hospital for psychiatric assessment. The evaluation showed acute suicidality in a patient with no social support, escalating hopelessness and frustration with her psychiatric care. Her symptoms and history raised the suspicion of a borderline personality disorder. At the time, however, this diagnosis had not been confirmed or documented. She voluntarily agreed to be admitted to our psychiatric hospital. The following day – because she was still suffering from acute suicidality – after receiving written informed consent she was screened for, and enrolled, in the KETA pilot study. The aim of the study was to treat suicidality specifically, taking into account that these patients may or may not have other, concurrent, psychiatric conditions. Although trying to stay as close to clinical reality as possible, we were very mindful of risk. Therefore, we critically evaluated and discussed the potential hazards for each individual patient before deciding whether that patient could participate. In the case of this subject, we consulted the physician who had treated her since March 2021. No past substance abuse was reported by this physician. Nevertheless, due to the setting and focus of the study on acute suicidality, there was no time available for extensive diagnostic trajectories. We acknowledge that ketamine is not an approved indication for suicidality, and this study explicitly explores its potential for this new patient group, which was also the reason for performing a pilot study before starting with a larger RCT.

When the patient enrolled in the KETA study, her MADRS and BSSI scores at baseline were 37 and 35, respectively; 1 h post-intervention these had reduced to 31 and 29, and to 32 and 25, respectively at 1 day following the intervention. After 1 week, at the end of follow-up, these had increased back to 36 and 31, respectively. These scores indicated a rapid but transient reduction in both depression symptoms and suicidal ideation.

Post-intervention, the patient attributed the transient reduction in suicidal ideation to two factors: a positive experience from the direct psychoactive effects of ketamine and an abrupt diminution of her persistent intrusive and obsessive thoughts, which she described as ‘the intrusive thoughts being pushed to the side of her head’. Around 2 h after receiving the intranasal spray, the intrusive thoughts returned. During the subsequent follow-up period of 1 week, the patient returned to her sheltered housing facility.

Over the following weeks, the patient wanted to start consuming ketamine recreationally. For the first time she came into contact with a drug dealer, who provided her with ketamine. This became apparent to her psychiatric clinicians after approximately 4 weeks. She intranasally used illicitly obtained ketamine in powdered form, in daily doses reaching up to approximately 1 g. Given its illicit source, the specific type (S-ketamine, R-ketamine or racemic mixture) and purity were unknown. She reported no adverse urinary, cognitive or other effects. She described ketamine craving in the form of longing for the calming effect on her intrusive thoughts, which she had first experienced during study participation. Furthermore, she liked the visual effects and sensations caused by ketamine, such as having the feeling that her arms and legs were several metres long. Because of its subjective effect on time perception, she generally used ketamine throughout the day to make time pass faster. If she had to go outside, she started to take stimulants such as cocaine or 3-methylmethcathinone (3-MMC) to reduce the effects of ketamine. She obtained this drug via the dealer, who presented her with a list of the different drugs that were available. When she returned home, she felt a need to counter the effect of the stimulants in turn with ketamine, resulting in a loop of stimulant and ketamine use. She required increasing doses to achieve these effects. Eventually her drug abuse caused financial problems. Subsequently this resulted in an inability to acquire more ketamine, and her sheltered housing facility pressured her to quit using ketamine because she would probably be evicted if she continued abusing it. A subsequent suicide attempt resulted from this increasing stress and her inability to obtain more ketamine, even though she did not experience physical withdrawal symptoms.

Although the patient had no prior history of addiction, and only sporadically used alcohol and cannabis, referral and treatment of her addiction were strongly advised after the discovery of her illicit abuse. The patient chose not to follow this advice.

The threat of eviction from her home caused her to stop abusing ketamine and other drugs. When asked what had caused this sudden ability to stop, she mentioned that ‘her autism helps her to really go for it when she has made a decision’. She also mentioned that the interference of ketamine abuse with her current treatment, the threat of losing her place at the sheltered living facility and financial problems were the main motivators to stop, because she realised that ketamine abuse was causing more harm than good. Unfortunately, the patient later relapsed into ketamine and cocaine addiction.

## Background

Ketamine has been shown to have a rapid and positive effect on depressive symptoms and suicidality.^3–6^ Moreover, it has been suggested that ketamine’s effect on suicidality may be independent of an improvement in depressive symptoms.^7^ Given its ability to rapidly reduce depressive symptoms and its potential effectiveness for alleviating suicidal symptoms, ketamine may present a novel treatment for acute suicidality. To test the hypothesis that ketamine could have an antisuicidal effect, irrespective of the underlying diagnosis, we designed the KETA study and performed the KETA pilot beforehand. The protocol for the KETA study has been published elsewhere.^8^

## Abuse liability of ketamine

Despite these potential advantages, ketamine treatment carries a well-known risk for addiction. Both intranasal and intravenous treatments have a rapid absorption rate, which increases abuse liability.^9^ This potential abuse liability is further complicated by ketamine often being available through illicit means outside of medical control. Furthermore, preclinical evidence shows that dose escalation is needed to retain the same effect with repeated use, with S-ketamine having a higher addiction liability compared with R-ketamine.^10^ A 2015 case report by Bonnet, for the first time, shed light on ketamine abuse following intended clinical use. That article presents the case of a 50-year-old nurse with depression who, in an attempt to alleviate her symptoms, began intramuscularly injecting herself with ketamine that she had taken from hospital supplies. It needs to be taken into account that in this case the ketamine was not prescribed by a doctor, and thus was not properly monitored. Notwithstanding, this work provided a first signal of the abuse potential of ketamine when administered for the treatment of a mood disorder.

Despite this addiction liability, a recent scoping review found that single or repeated ketamine administrations in controlled settings did not cause misuse, dependence or gateway activity in patients with treatment-resistant depression. It should, however, be noted that most studies did not systematically evaluate abuse liability via validated scales or questionnaires, which probably led to underreporting of these adverse events.^11–14^ Given the fact that ketamine addiction can have severe detrimental effects on the brain, reporting of abuse development in clinical settings is very important.^15^ Besides neurotoxic effects, ketamine can also devastatingly impact urological health, with prevalence rates of lower urinary tract symptoms in 44–77% of ketamine abusers and 8–30% for upper urinary tract disease.^16^ Although one may not directly draw conclusions from the abuse liability of recreational ketamine for clinical therapy, literature on this topic may provide insights. Recreational ketamine imposes a significant burden on society. In 2008/2009 the prevalence of ketamine abuse in the UK was 1.7%, with a lifetime prevalence of 4%,^17,18^ and ketamine abuse was around 1% in American college students.^19^ Furthermore, recreative ketamine use doubled in the UK between 2016 and 2025. We are aware that treating complex, multimorbid subjects may pose additional risk. Although not all of this has reached the medical literature, cases such as that of Matthew Perry painfully illustrate that risk.^20^ However, given the fact that ketamine may represent a promising treatment even for severely mentally ill multimorbid patients, it is surmisable that, in real-world clinical settings, these patients may receive ketamine treatment.

Further exploration into patient-specific characteristics and dosing strategies is necessary to further optimise treatment and hopefully reduce the likelihood of addiction occurring as a result of ketamine treatment for a mental disorder.^21^ Several sources postulate that intranasal ketamine is absorbed by the olfactory nerve,^22,23^ which may in turn lead to its addictive potential.

Furthermore, recent work has shed light on self-medication with ketamine; that study based its conclusions on the Global Drug Survey (GDS).^24^ The authors used a model that predicted level of use among several demographic groups. They inferred that yearly ketamine use could be as high as 33.3 g for males and 29.46 g for females. These doses far exceed clinically used dosing regimens and may lead to serious health consequences. This leads to the question of whether clinicians prescribing ketamine should take into account such contextual information when evaluating the risk of abuse. This in turn presents the dilemma of whether to prescribe ketamine to subjects who have used it in the past and who might benefit from it, but who may also be at increased risk of developing ketamine abuse.

## Implications for further research and treatment

This adverse outcome of ketamine treatment for acute suicidality led us to the decision to implement a number of amendments to the protocol of the subsequent RCT. The case was discussed in a departmental research meeting, and subsequently with the IRB and Data Safety Monitoring Board overseeing the KETA study. The main goal was to find ways to warrant the safety of future study participants and patients undergoing ketamine treatment. The following considerations were addressed.

Inclusion and exclusion criteria: in the protocol for this pilot study, patients with a history of ketamine or phencyclidine addiction were excluded from participation. We subsequently debated whether we should also exclude patients with any other form of substance abuse or addiction in the past or present. However, the patient presented here had no prior history of any substance abuse and would therefore not have been excluded. Furthermore, patients with substance (e.g. alcohol) abuse may also suffer from suicidal ideation and could possibly benefit from ketamine treatment.^25^ Excluding this group would have limited the study’s ability to investigate the general effectiveness of ketamine on suicidality. However, treatment may also harm subjects if they develop substance abuse as a result of it. Current contraindications for intranasal ketamine treatment for depression are a past history of cerebral haemorrhage, aneurysmatic vascular disease or malformations and hypersensitivity for ketamine. Although the Spravato monograph mentions substance abuse and addiction as risk factors for ketamine abuse, it is not considered a contraindication for treatment. However, it may be that future studies will find otherwise, which again highlights the importance of systematic monitoring of abuse post-treatment.

Follow-up period: we increased the follow up period from 7 days to 4 weeks, in order to be better able to observe emerging ketamine addictions following treatment. A second amendment is that researchers enquire about signs of ketamine craving or abuse at all follow-up periods, and request that patients report craving or abuse to their treating psychiatrists.

A member of the study team with clinical experience is always present during the first hour post-intervention to help subjects with any distress they experience. This could also facilitate the identification of drug-liking

In summary, future studies on ketamine should evaluate abuse liability in light of the study medication and treatment regimen, patient group and inclusion and exclusion criteria. Monitoring during study treatment and prolonged follow-up of at least 1 month are important in identifying signs of drug craving, liking or abuse, either via direct enquiry or instruments such as the Ketamine Side Effect Tool (KSET).^26^ In addition, researchers should inform treating psychiatrists about the risks and signs of abuse or addiction and encourage them to actively inquire about these signs.

Ketamine holds promise as a powerful, rapid-acting and effective treatment for depression,^6^ and possibly for other psychiatric disorders, but it is not without risks, as evidenced by the case presented here. Although the risks associated with sub-anaesthetic dosages (<0.5 mg intravenously or oral/intranasal equivalent dose) appear to be moderate, those associated with ketamine abuse may cause substantial harm to the urinary tract, liver and brain.^15,27^ What is more, this case suggests that patients may still be at risk for abuse even without a prior history of substance abuse. Monitoring of possible abuse or addiction in all patients receiving ketamine treatment is essential to improve safety, and to provide the necessary help for patients who do experience craving and/or tendencies towards addiction development.

## Data Availability

The data are not publicly available due to restrictions pertaining to information that could compromise the privacy of the patient described in this case report. However, the data supporting the findings of this study are available on reasonable request from the corresponding author, J.F.M.S.

## References

[1] Roelandt GHJ , Strous JFM , Marijnissen RM , Kamphuis J , van Dalfsen JH , Schoevers RA. Single fixed-dose intranasal racemic ketamine treatment for the treatment of acute suicidality in a transdiagnostic patient population: results of a pilot study. Psychiatry Res [Epub ahead of print] 16 Dec 2026. Available from: 10.1016/j.psychres.2025.116909. 41456567

[2] WMA Declaration of Helsinki. Ethical Principles for Medical Research Involving Human Participants . World Medical Association (WMA), 2024 (https://www.wma.net/policies-post/wma-declaration-of-helsinki/). 10.1001/jama.2024.21972 39425955

[3] Witt K , Potts J , Hubers A , Grunebaum MF , Murrough JW , Loo C , et al. Ketamine for suicidal ideation in adults with psychiatric disorders: a systematic review and meta-analysis of treatment trials. Aust N Z J Psychiatry 2020; 54: 29–45. 31729893 10.1177/0004867419883341

[4] Domany Y , Shelton RC , Mccullumsmith CB. Ketamine for acute suicidal ideation. An emergency department intervention: a randomized, double-blind, placebo-controlled, proof-of-concept trial. Depress Anxiety 2020; 37: 224–33. 31733088 10.1002/da.22975

[5] Abbar M , Demattei C , El-Hage W , Llorca PM , Samalin L , Demaricourt P , et al. Ketamine for the acute treatment of severe suicidal ideation: double blind, randomised placebo controlled trial. BMJ 2022; 376: e067194. 35110300 10.1136/bmj-2021-067194 PMC8808464

[6] Marcantoni WS , Akoumba BS , Wassef M , Mayrand J , Lai H , Richard-Devantoy S , et al. A systematic review and meta-analysis of the efficacy of intravenous ketamine infusion for treatment resistant depression: January 2009 – January 2019. J Affect Disord 2020; 277: 831–41. 33065824 10.1016/j.jad.2020.09.007

[7] Wilkinson ST , Ballard ED , Bloch MH , Mathew SJ , Murrough JW , Feder A , et al. The effect of a single dose of intravenous ketamine on suicidal ideation: a systematic review and individual participant data meta-analysis. Am J Psychiatry 2018; 175: 150–8. 28969441 10.1176/appi.ajp.2017.17040472 PMC5794524

[8] Strous JFM , Roelandt GHJ , van Dalfsen JH , Kamphuis J , Marijnissen RM , Schoevers RA. The Ketamine Trial for Acute Suicidality (KETA): study protocol of a double-blind randomized placebo-controlled superiority trial on intranasal racemic ketamine compared to the active placebo intranasal midazolam as treatment for acute suicidality. Int J Methods Psychiatr Res 2025; 34: e70044. 41255293 10.1002/mpr.70044 PMC12627964

[9] Farré M , Cami J. Pharmacokinetic considerations in abuse liability evaluation. Br J Addict 1991; 86: 1601–6. 1786493 10.1111/j.1360-0443.1991.tb01754.x

[10] Bonaventura J , Lam S , Carlton M , Boehm MA , Gomez JL , Solís O , et al. Pharmacological and behavioral divergence of ketamine enantiomers: implications for abuse liability. Mol Psychiatry 2021; 26: 6704–22. 33859356 10.1038/s41380-021-01093-2 PMC8517038

[11] Breeksema JJ , Kuin BW , Kamphuis J , van den Brink W , Vermetten E , Schoevers RA. Adverse events in clinical treatments with serotonergic psychedelics and MDMA: a mixed-methods systematic review. J Psychopharmacol 2022; 36: 1100–17. 36017784 10.1177/02698811221116926 PMC9548934

[12] Le TT , Cordero IP , Jawad MY , Swainson J , Di Vincenzo JD , Jaberi S , et al. The abuse liability of ketamine: a scoping review of preclinical and clinical studies. J Psychiatr Res 2022; 151: 476–96. 35623124 10.1016/j.jpsychires.2022.04.035

[13] Chubbs B , Wang J , Archer S , Chrenek C , Khullar A , Wolowyk M , et al. A survey of drug liking and cravings in patients using sublingual or intranasal ketamine for treatment resistant depression: a preliminary evaluation of real world addictive potential. Front Psychiatry 2022; 13: 1016439. 36465297 10.3389/fpsyt.2022.1016439 PMC9714431

[14] Acevedo-Diaz EE , Cavanaugh GW , Greenstein D , Kraus C , Kadriu B , Zarate CA , et al. Comprehensive assessment of side effects associated with a single dose of ketamine in treatment-resistant depression. J Affect Disord 2020; 263: 568–75. 31791675 10.1016/j.jad.2019.11.028 PMC8457026

[15] Strous JFM , Weeland CJ , van der Draai FA , Daams JG , Denys D , Lok A , et al. Brain changes associated with long-term ketamine abuse, a systematic review. Front Neuroanat 2022; 16: 795231. 35370568 10.3389/fnana.2022.795231 PMC8972190

[16] Andrade C. Ketamine-associated uropathy during therapeutic and nontherapeutic use: prevalence, clinical features, mechanisms, and strategies for risk reduction. J Clin Psychiatry 2025; 86: 25f16083. 10.4088/JCP.25f16083 40965833

[17] Zou J , Tan S. Emerging trends in the abuse of ketamine and its side effects on health: toxicology and addiction potential. Adv Clin Toxicol 2016; 1: 000105.

[18] Hoare WJ , Moon D. Drug Misuse Declared: Findings from the 2009/10 British Crime Survey . Home Office Statistical Bulletin, 2010 (https://assets.publishing.service.gov.uk/media/5a7aae3fed915d71db8b1bf5/hosb1310.pdf).

[19] Maxwell JC. Party drugs: properties, prevalence, patterns, and problems. Subst Use Misuse 2005; 40: 1203–40. 16048814 10.1081/JA-200066736

[20] Stevens M , Hamby C . Matthew Perry’s Death Shines a Harsh Light on Ketamine Treatment . The New York Times, 2024 (https://www.nytimes.com/2024/08/19/arts/television/matthew-perry-ketamine-treatment.html).

[21] Hochschild A , Grunebaum MF , Mann JJ. The rapid anti-suicidal ideation effect of ketamine: a systematic review. Prev Med 2021; 152: 106524. 34538369 10.1016/j.ypmed.2021.106524

[22] Qiu Y , Huang S , Peng L , Yang L , Zhang G , Liu T , et al. The nasal–brain drug delivery route: mechanisms and applications to central nervous system diseases. MedComm 2025; 6: e70213. 40487748 10.1002/mco2.70213 PMC12141948

[23] Andrade C. Intranasal drug delivery in neuropsychiatry: focus on intranasal ketamine for refractory depression. J Clin Psychiatry 2015; 76: e628–31. 26035196 10.4088/JCP.15f10026

[24] Smith G , Piatkowski T , Ferris J , Bonenti B , Davies E , Barratt MJ , et al. Self-treatment of psychiatric conditions using ketamine: patterns, characteristics, and retrospective insights. J Psychopharmacol [Epub ahead of print] 22 Oct 2025. Available from: 10.1177/02698811251378509. PMC13351112 41122942

[25] Holmstrand C , Bogren M , Mattisson C , Brådvik L. Long-term suicide risk in no, one or more mental disorders: the Lundby Study 1947-1997. Acta Psychiatr Scand 2015; 132: 459–69. 26402416 10.1111/acps.12506 PMC5054879

[26] Bayes A , Short B , Zarate CA , Park L , Murrough JW , McLoughlin DM , et al. The Ketamine Side Effect Tool (KSET): a comprehensive measurement-based safety tool for ketamine treatment in psychiatry. J Affect Disord 2022; 308: 44–6. 35405177 10.1016/j.jad.2022.04.020 PMC9133168

[27] Orhurhu VJ , Vashisht R , Claus LE , Cohen SP. Ketamine Toxicity . StatPearls, 2023 (https://www.ncbi.nlm.nih.gov/books/NBK541087/). 31082131

